# Supplementary material for: The cryo-EM structure of bacteriophage PRR1 and its role in conjugation inhibition
Source: J Virol. 2026 Jul 10;100(8):e00515-26. doi: 10.1128/jvi.00515-26 (PMC13483237; doi:10.1128/jvi.00515-26)
Supplement: Supplementary file 1 [file jvi.00515-26-s0001.pdf]

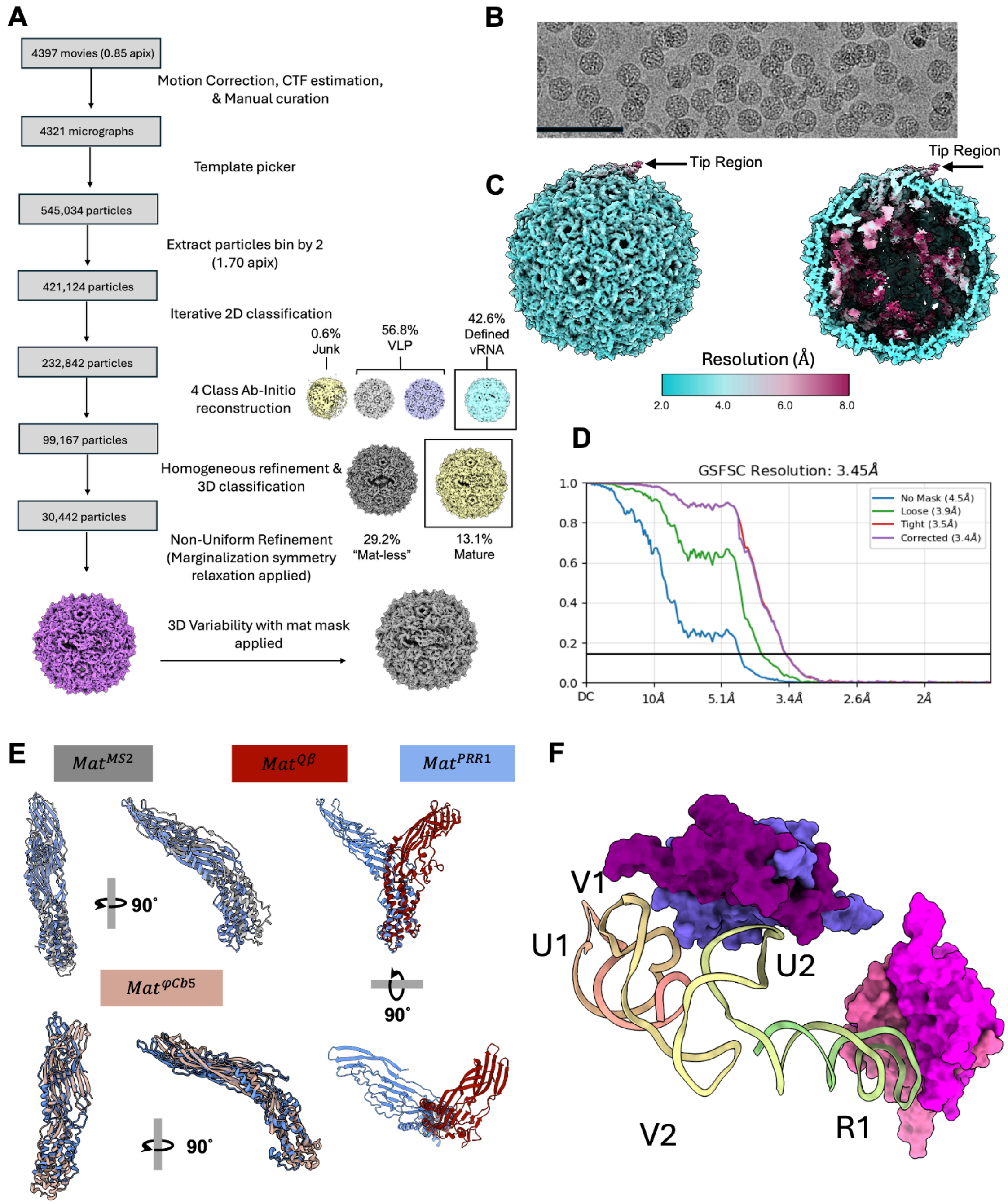

A
4397 movies (0.85 apix)
Motion Correction, CTF estimation, & Manual curation
4321 micrographs
Template picker
545,034 particles
Extract particles bin by 2 (1.70 apix)
421,124 particles
Iterative 2D classification
232,842 particles
0.6% Junk
56.8% VLP
42.6% Defined vRNA
4 Class Ab-Initio reconstruction
99,167 particles
Homogeneous refinement & 3D classification
30,442 particles
Non-Uniform Refinement (Marginalization symmetry relaxation applied)
29.2% "Mat-less"
13.1% Mature
3D Variability with mat mask applied
B
C
Tip Region
Resolution (Å)
2.0
4.0
6.0
8.0
D
GSFSC Resolution: 3.45Å
No Mask (4.5Å)
Loose (3.9Å)
Tight (3.5Å)
Corrected (3.4Å)
DC
10Å
5.1Å
3.4Å
2.6Å
2Å
E
Mat^MS2
Mat^Qβ
Mat^PRR1
Mat^φCb5
90˚
F
V1
U1
U2
V2
R1

Figure S1. Data processing of PRR1 and analysis of Mat, vRNA, and Coat.

(A) The workflow performed in cryoSPARC on the vitrified PRR1 virions. *Ab initio* reconstruction resulted in four classes: junk (yellow), empty VLP 1 (gray), empty VLP 2 (purple), and particles with defined vRNA density (blue, boxed). The ~99k particles contributed to the blue map with defined vRNA density were further classified into two classes: “Mat-less” (gray) and mature (yellow, boxed). The ~30k mature particles were further processed as shown.

(B) An example micrograph of the vitrified PRR1 virions. The black scale bar (100 nm) is shown for reference.

(C) The local resolution of the electron density of the PRR1 virion estimated in cryoSPARC.

(D) The Gold Standard Fourier Shell Correlation (GSFSC) of the cryo-EM reconstruction for the mature PRR1 virion.

(E) Structural comparisons of the Mat proteins from MS2, Q□, and ϕCb5 with $\mathrm{Mat}^{\mathrm{PRR1}}$. Each Mat is depicted in the color that its name is boxed in. The PDB accession codes for each Mat are: MS2 (6NM5), Q□ (5MNT), and ϕCb5 (8UCR).

(F) The 3′ vRNA of PRR1 interacts with two Coat dimers. Loops U2 and R1 (vRNA) each interact with a Coat dimer (purple and slate purple Coat dimer binding U2; pink and magenta Coat dimer binding R1).

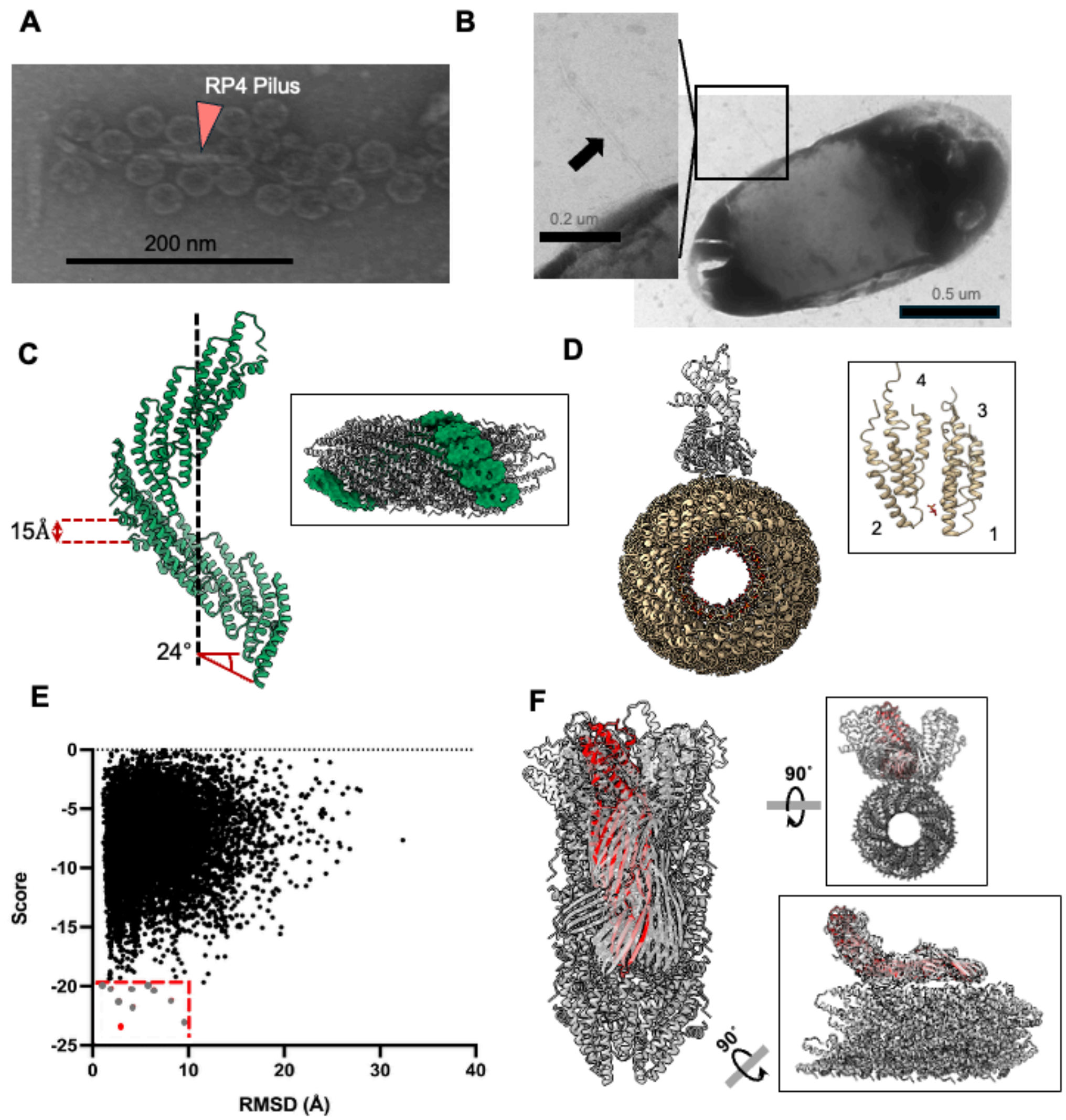

A
RP4 Pilus
200 nm
B
0.2 um
0.5 um
C
15Å
24°
D
4
3
2
1
E
0
-5
-10
-15
-20
-25
Score
0
10
20
30
40
RMSD (Å)
F
90˚
90˚

Figure S2. Analysis of the RP4 pilus.
(A) Negative-stain TEM image showing PRR1 particles (spherical particles) bound to the RP4 pilus (filamentous indicated by an orange arrow).
(B) XL1-Blue (RP4) cells producing RP4 pili. The inset shows a zoomed-in view of the pilus (black arrow). Pili were observed at a rate of about 1 per every 10th cell.
(C) A full turn of the RP4 pilus. The axis running down the center of the pilus is shown as a black dotted line. This was used to measure the twist (angle between adjacent pilin): 24°. The rise (vertical distance between adjacent pilin) was 15 Å. The inset shows the full turn (green, surface representation) in the context of the rest of the pilus (gray).
(D) The Mat^{MS2} (gray) bound to the F-pilus (tan): (pdb: 6NM5). The inset shows the lumen of the F-pilus contains a phospholipid (red) surrounded by 4 pilin monomers.
(E) Plot of the 10,000 Mat^{PRR1}–RP4 pilus structures from the docking study. Root mean square deviation (RMSD) values were calculated relative to the starting structure based on the MS2–F pilus complex (PDB: 6NM5), reflecting Mat structural homology. The red, boxed region indicates the 10 best-scored models. The best model is colored red, while the others are colored gray.
(F) A structural alignment of the 10 best-scored models. The best model is highlighted in red, while the remaining models are colored transparent gray. The insets show front and side views of the structures.

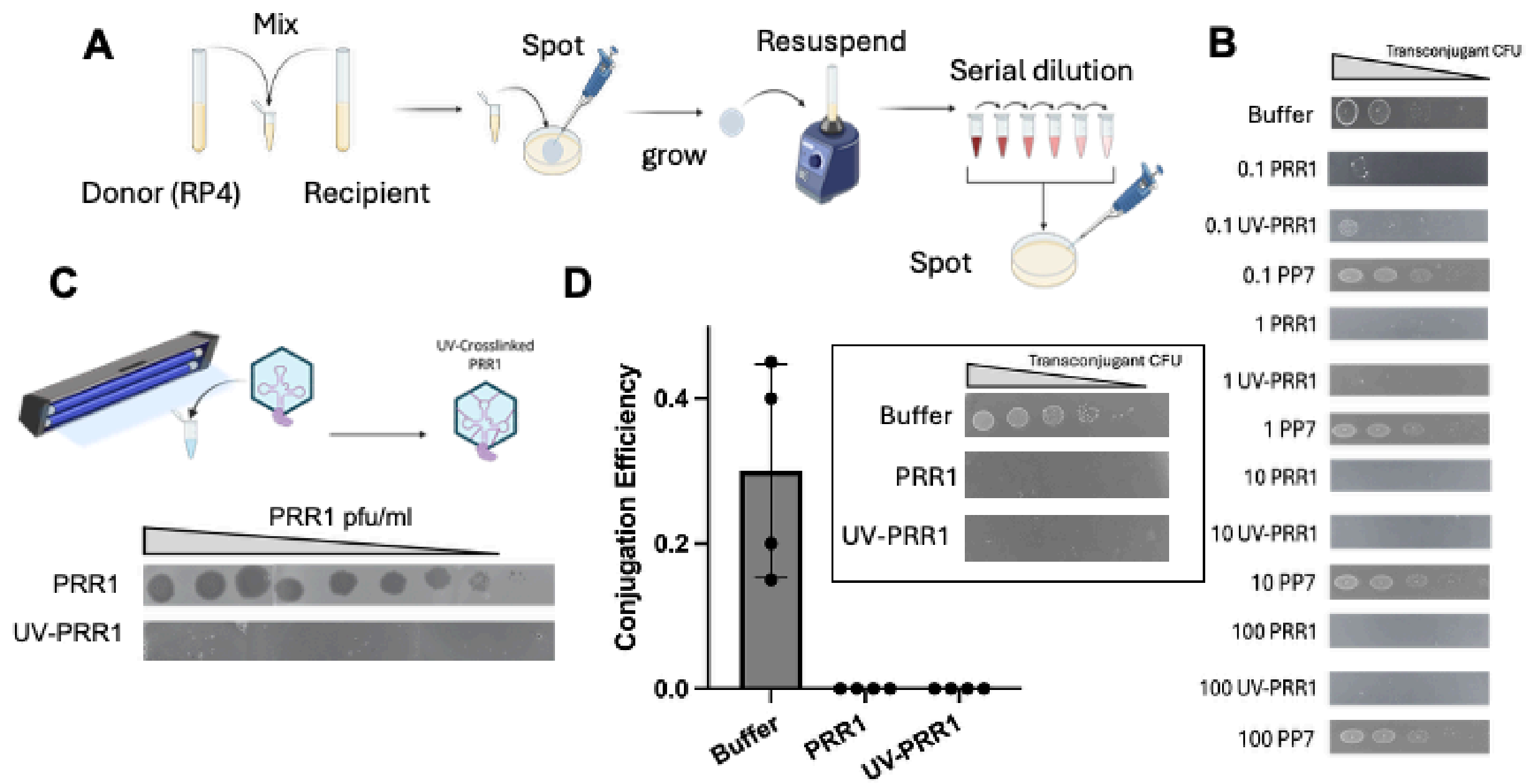

A
Mix
Donor (RP4)
Recipient
Spot
grow
Resuspend
Serial dilution
Spot
B
Transconjugant CFU
Buffer
0.1 PRR1
0.1 UV-PRR1
0.1 PP7
1 PRR1
1 UV-PRR1
1 PP7
10 PRR1
10 UV-PRR1
10 PP7
100 PRR1
100 UV-PRR1
100 PP7
C
UV-Crosslinked PRR1
PRR1 pfu/ml
PRR1
UV-PRR1
D
Conjugation Efficiency
0.4
0.2
0.0
Buffer
PRR1
UV-PRR1
Transconjugant CFU
Buffer
PRR1
UV-PRR1

Figure S3. Data of the conjugation assays with PRR1 and UV-PRR1.

(A) Schematic illustrating the experimental strategy used to measure RP4 conjugation efficiency. We mixed the donor and recipient cells in the presence of buffer, PRR1, UV-PRR1, or PP7, respectively, allowing them to grow on non-selective media for 2.5 hours. Then the cells were resuspended, diluted, and spotted on doubly selective media (see Methods) to allow only recipients that received the RP4 plasmid to grow.

(B) Images from the conjugation assay with PAO1Δ*pilA* (RP4) visualized on plates. The panel shows the result of the conjugation assay after adding different MOIs (from 0.1 to 100) of PRR1, UV-PRR1, PP7, or buffer.

(C) A schematic detailing the UV-irradiation procedure. PRR1 was aliquoted into tubes and exposed to UV-light for an hour. UV-induced cross-links between the vRNA and the Coat (shown as lines) restrict the vRNA to the capsid. The titer of PRR1 on PAO1Δ*pilA* (RP4) cells before (WT-PRR1) and after (UV-PRR1) exposure to UV light.

(D) The effect of PRR1, UV-PRR1, and buffer at an MOI=1 on RP4 conjugation in *E. coli*. Each point represents the average of n=4 replicates. The inset shows representative plates from the conjugation assay using the *E. coli* strain XL1-blue (RP4). The MOIs of PRR1 and UV-PRR1 were calculated to be 1.

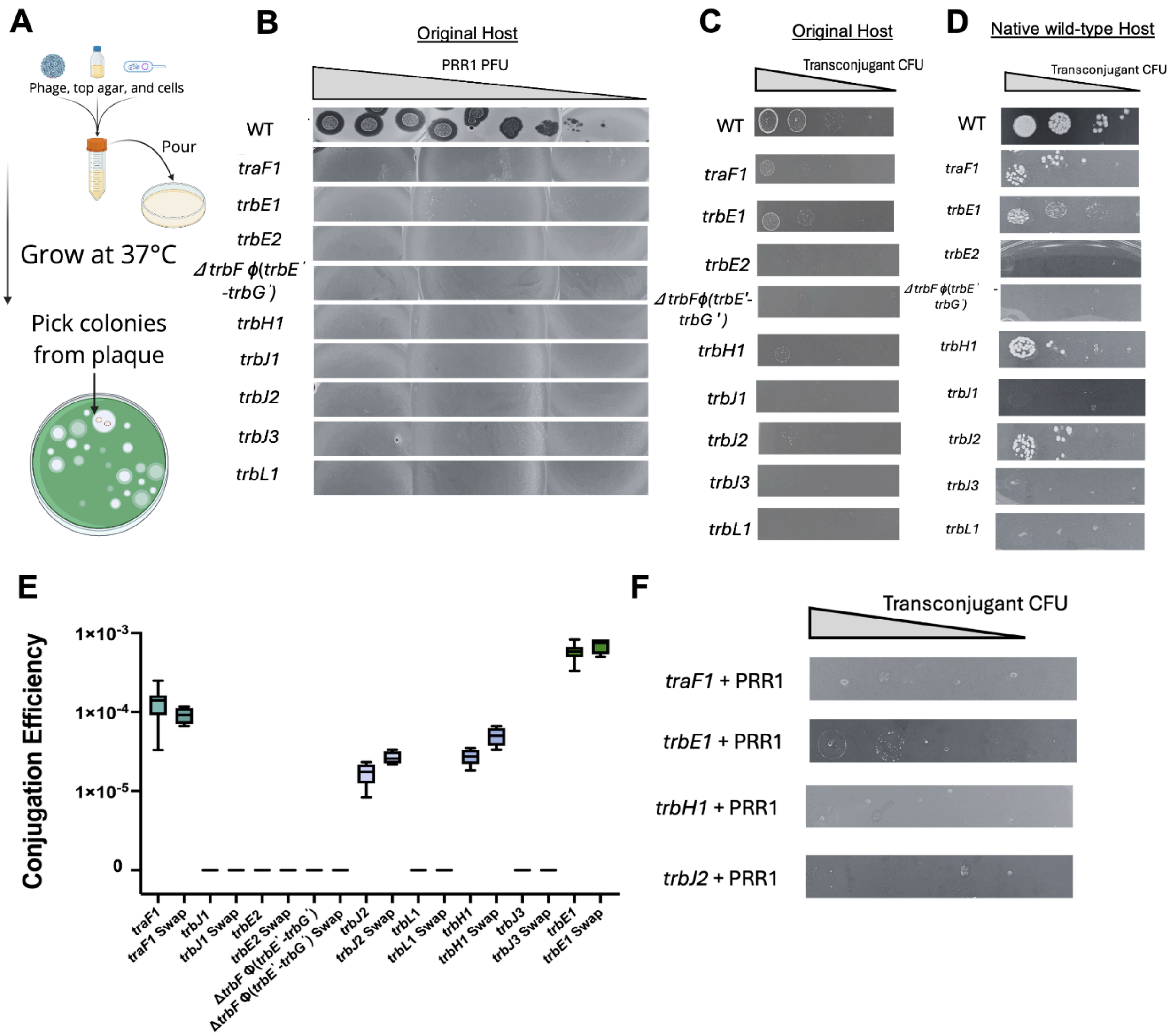
A
Phage, top agar, and cells
Pour
Grow at 37°C
Pick colonies from plaque
B
Original Host
PRR1 PFU
WT
traF1
trbE1
trbE2
ΔtrbF ϕ(trbE′-trbG′)
trbH1
trbJ1
trbJ2
trbJ3
trbL1
C
Original Host
Transconjugant CFU
D
Native wild-type Host
Transconjugant CFU
E
Conjugation Efficiency
1×10^-3
1×10^-4
1×10^-5
0
traF1
traF1 Swap
trbJ1
trbJ1 Swap
trbE2
trbE2 Swap
ΔtrbF Φ(trbE′-trbG′)
ΔtrbF Φ(trbE′-trbG′) Swap
trbJ2
trbJ2 Swap
trbL1
trbL1 Swap
trbH1
trbH1 Swap
trbJ3
trbJ3 Swap
trbE1
trbE1 Swap
F
Transconjugant CFU
traF1 + PRR1
trbE1 + PRR1
trbH1 + PRR1
trbJ2 + PRR1

Figure S4. Conjugation assays of the mutant RP4 cells.

(A) The strategy for generating PRR1 and antibiotic resistant colonies. The cells and phage were plated on triple-selective media (see Methods) to preserve RP4. Cells were allowed to grow for 2 days, and resistant colonies were picked from within the PRR1 plaques.

(B) An image of the spot titer for each RP4 mutant generated from the dual selection assay. Each mutant lacks PRR1 plaques, demonstrating their resistance.

(C) An image of the original mutant conjugation assay visualized on plates without PRR1 addition.

(D) The plates from the conjugation assay for each host swapped mutant.

(E) All replicates of **(E)** graphed next to their original host counterpart. The p-values calculated for the mutants that were able to transfer were 0.7866, 0.9994, 0.9839, and 0.0507 for *traF1*, *trbJ2*, *trbH1*, and *trbE1* respectively (significance was determined using an ordinary one-way ANOVA with Dunnett's multiple comparisons test). Each bar is an average of n=4 replicates.

(F) An image of plates from the conjugation assay using the four mutants that can transfer, with the addition of PRR1 at an MOI of 10.

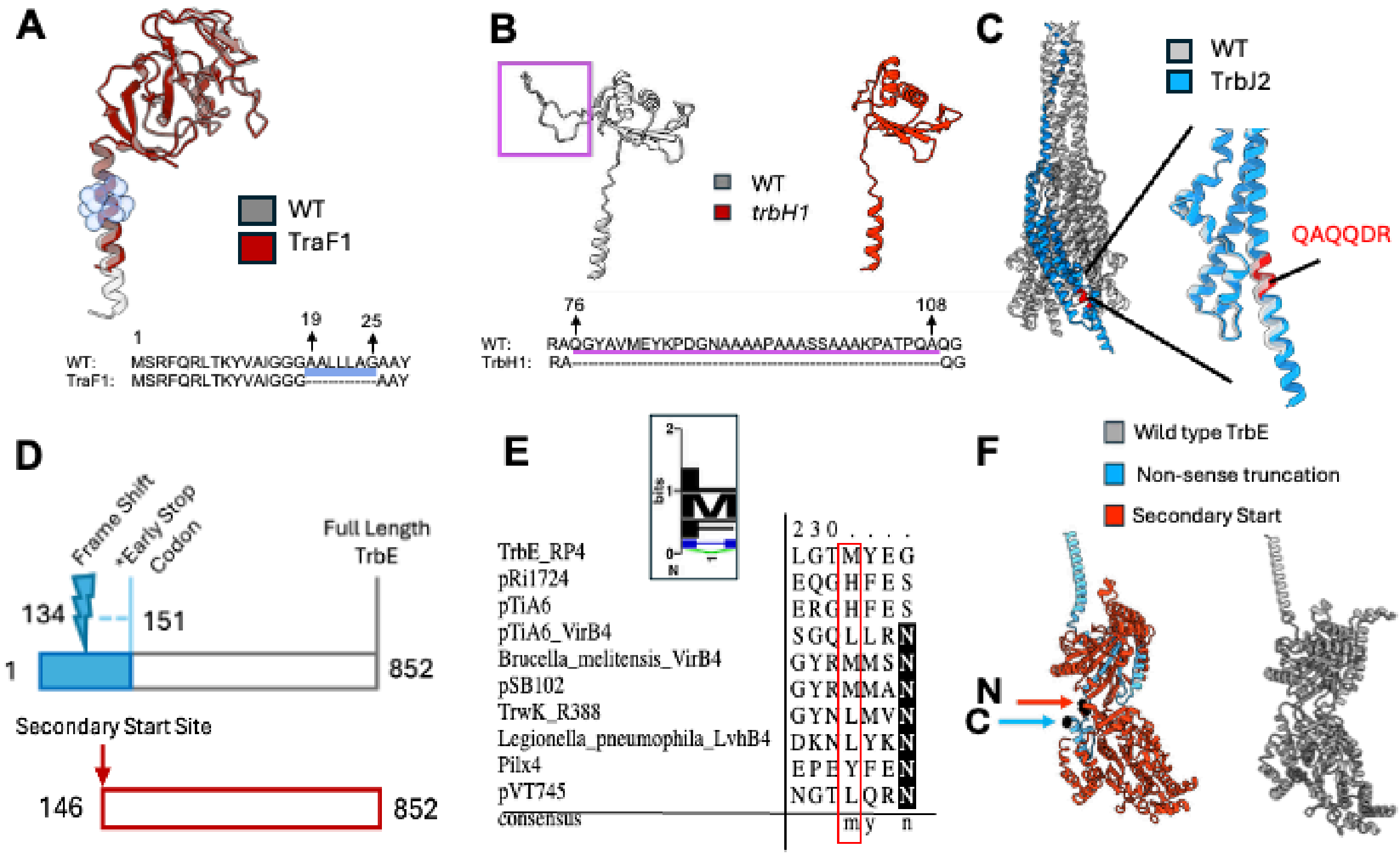
A
WT
TraF1
1
19
25
WT: MSRFQRLTKYVAIGGGAALLLAGAAY
TraF1: MSRFQRLTKYVAIGGG------------AAY
B
WT
trbH1
76
108
WT: RAQGYAVMEYKPDGNAAAAPAAASSAAAKPATPQAQG
TrbH1: RA---QG
C
WT
TrbJ2
QAQQDR
D
Frame Shift
+Early Stop Codon
Full Length TrbE
134
151
1
852
Secondary Start Site
146
852
E
bits
TrbE_RP4
pRi1724
pTiA6
pTiA6_VirB4
Brucella_melitensis_VirB4
pSB102
TrwK_R388
Legionella_pneumophila_LvhB4
Pilx4
pVT745
consensus
F
Wild type TrbE
Non-sense truncation
Secondary Start
N
C

Figure S5. In-depth look at the four mutants that retain the ability to transfer the RP4 plasmid.
(A) The *traF1* mutant protein (red) contained a 7 amino acid deletion (ΔA19-G25) in its transmembrane helix. The deleted residues are shown in sphere form (blue) on the structure of the wild type TraF protein (gray), and underlined (blue) on the sequence alignment.
(B) The *trbH1* mutant (red) contained a 33 amino acid deletion (ΔQ76-A108) in a predicted flexible region. The deleted residues from the wild type TrbH protein are underlined in purple on the sequence alignment and boxed in purple on the structure of the wild type protein.
(C) The *trbJ2* mutant contained a 6 amino acid insertion (R234_Q235 ins QAQQDR) in an important helix for pentamerization and TrbL binding. A view of the wild type TrbJ pentamer (gray) with a single TrbJ2 mutant monomer inserted (blue). The zoomed-in panel shows the mutant has an extended helix due to the addition of 6 amino acids (red). The typical length of the TrbJ protein is shown by the wild type protein (gray transparent).
(D) The *trbE1* mutant contained a 10 base pair deletion that results in a premature stop codon at position 151, 18 amino acids downstream of where the mutation lies (position 134). However, an alternative start codon is present at position 146, which translates the remaining portion of the TrbE protein.
(E) The alignment of several homologs of TrbE highlights the fact that there is a conserved internal start codon downstream of the full length product (boxed in red). The inset shows a conservation plot at the position of the secondary start site.
(F) The predicted structure of the heterodimer formed by the non-sense truncation product (blue) and the secondary start site product (red). A wild type TrbE monomer (gray) is shown for reference.

Table S1. Conjugation efficiencies of transfer-positive RP4 mutants relative to wild type. Calculations for conjugation efficiency are described in the methods section.

Strain	Conjugation Efficiency	Percent Reduction (from WT)	Percent Reduction (from WT with additional PRR1)
WT	0.210%	-	-
traF1	0.0136%	93.5%	100%
trbE1	0.0583%	72.2%	94.9%
trbH1	0.00275%	98.7%	100%
trbJ2	0.00171%	99.2%	99.7%

Table S2. Bacterial and viral strains, and plasmids used.

1. Bacterial and viral strains	Description	References
BL21(DE3)pLysS (pRSF_PRR1)	Expression of ssRNA Phage *Perrunavirus olsenii cDNA*	This study
P. aeruginosa PAO1Δ*pilA* (RP4)	pilA-, contains RP4	This study
P. aeruginosa PAO1Δ*pilA* (pUCP19_GmR)	pilA-, Gentamicin resistant on pUCP19	This study
E. coli XL1-Blue (RP4)	Contains the RP4 plasmid	This study
E. coli XL1-Blue (pUCP19_CmR)	Contains chloramphenicol resistance on pUCP19	This study
P. aeruginosa PAO1Δ*pilA* (RP4*ΔtrbC)*	pilA-, RP4 TrbC-	This study
P. aeruginosa PAO1Δ*pilA* (RP4Δ*trbC)* (pUCP19_*trbC*_ variant)	pilA-, RP4 TrbC-, pUCP19 containing one of the 15 TrbC variants or lacking the TrbC gene	This study
P. aeruginosa PAO1Δ*pilA* (RP4_ *traF1*)	pilA-, RP4 mutant *traF1*	This study
P. aeruginosa PAO1Δ*pilA* (RP4_ *traE1*)	pilA-, RP4 mutant *trbE1*	This study
P. aeruginosa PAO1Δ*pilA* (RP4_ *trbE2*)	pilA-, RP4 mutant *trbE2*	This study
P. aeruginosa PAO1Δ*pilA* (RP4_Δ*trbFϕ(trbE'-trbG')*)	pilA-, RP4 mutant Δ*trbFϕ(trbE'-trbG')*	This study
P. aeruginosa PAO1Δ*pilA* (RP4_ *trbH1*)	pilA-, RP4 mutant *trbH1*	This study
P. aeruginosa PAO1Δ*pilA* (RP4_ *trbJ1)*	pilA-, RP4 mutant *trbJ1*	This study
P. aeruginosa PAO1Δ*pilA* (RP4_ *trbJ2*)	pilA-, RP4 mutant *trbJ2*	This study
P. aeruginosa PAO1Δ*pilA* (RP4_ *trbJ3*)	pilA-, RP4 mutant *trbJ3*	This study
P. aeruginosa PAO1Δ*pilA* (RP4_ *trbL1*)	pilA-, RP4 mutant *trbL1*	This study
**2. Plasmids**	**Description**	**References**
pRSF-PRR1	The cDNA of PRR1 cloned under the T7 promoter	This study
pUCP19	*P. aeruginosa* compatible pUC19	ATCC
pUCP19_*trbC*	Contains the wild-type TrbC protein	This study
pUCP19_*trbC*_S1A	Contains the S1ATrbC protein	This study
pUCP19_*trbC*_E2A	Contains the E2A TrbC protein	This study
pUCP19_*trbC*_T4A	Contains the T4A TrbC protein	This study

2. Plasmids Continued	Description	References
pUCP19_*trbC*_S7A	Contains the S7A TrbC protein	This study
pUCP19_*trbC*_E11A	Contains the E11A TrbC protein	This study
pUCP19_*trbC*_S12A	Contains the S12A TrbC protein	This study
pUCP19_*trbC*_W13A	Contains the W13A TrbC protein	This study
pUCP19_*trbC*_N16A	Contains the N16A TrbC protein	This study
pUCP19_*trbC*_N19A	Contains the N19A TrbC protein	This study
pUCP19_*trbC*_Q68A	Contains the Q68A TrbC protein	This study
pUCP19_*trbC*_N69A	Contains the N69A TrbC protein	This study
pUCP19_*trbC*_S72A	Contains the S72A TrbC protein	This study
pUCP19_*trbC*_R77A	Contains the R77A TrbC protein	This study
pUCP19_*trbC*_G78A	Contains the G78A TrbC protein	This study
pUCP19_GmR	Gentamicin resistant	This study
pUCP19_CmR	Chloramphenicol resistant	This study
RP4	The wild-type RP4 plasmid (IncP)	R. Young/CPT
RP4_*traF1*	RP4 containing a 7 amino acid deletion in TraF (19-25)	This study
RP4_*traE1*	RP4 containing a 10 base pair deletion in *trbE* (405-414)	This study
RP4_*trbE2*	RP4 containing a 4 amino acid deletion in TrbE(622-625)	This study
RP4_Δ*trbFϕ(trbE'-trbG')*	RP4 containing a deletion at residues 836-852 of TrbE, a full deletion of TrbF, and a deletion of residues 1-212 of TrbG; The protein sequence of TrbE contains no stop codon, so a fusion with the truncated TrbG is created	This study
RP4_*trbH1*	RP4 containing a deletion of 33 amino acids within TrbH (76-108)	This study
RP4_*trbJ1*	RP4 containing a 3 residue insertion between residues 238 and 239 (Ins QAQ)	This study
RP4_*trbJ2*	RP4 containing an insertion of 6 residues between R234 and Q235 (Ins QAQQDR)	This study
RP4_*trbJ3*	RP4 containing a 6 residue deletion within TrbJ (229-234)	This study

Table S3. Cryo-EM data collection, processing, refinement, and validation statistics.

PRR1	
Data Collection and Processing	
Voltage (kV)	300
Defocus Range (μm)	1.2 to 2.2
Pixel size (Å)	0.86
Electron exposure (e–/Å2)	50
Electron dosage per frame	1.5
Frame duration (sec/frame)	0.060
Exposure time (sec)	2.5
Slit size (eV)	15
Objective Aperture Size	100
Symmetry Imposed	C1
Micrographs Collected	4397
Final Particle Images (no.)	30,442
Reconstruction Software	cryoSPARC
Map Resolution (Å)	3.45
FSC Threshold	0.143
Refinement	
Initial Model Used (PDB code)	Coat Protein (2VF9)<br>Maturation Protein (AlphaFold Prediction)<br>RNA (this study)
Model Composition	
Chains	180
Total Atoms	192,164
Non-Hydrogen Atoms	184,466
Protein Residues	23,724

PRR1 Continued	
Refinement	
Model Composition	
Nucleotide Residues	132
Ligands	0
R.M.S. Deviations	
Bond Length (Å)	0.003
Bond Angles (°)	0.835
**Validation**	
MolProbity Score	2.69
Clash Score	33.68
Rotamer Outliers (%)	0.01
Ramachandran Plot	
Favored (%)	83.82
Allowed (%)	14.78
Outliers (%)	1.40
